# Liquid Chromatographic Methods for the Determination of Vildagliptin in the Presence of its Synthetic Intermediate and the Simultaneous Determination of Pioglitazone Hydrochloride and Metformin Hydrochloride

**Published:** 2011-09

**Authors:** Ramzia I. El-Bagary, Ehab F. Elkady, Bassam M. Ayoub

**Affiliations:** *Pharmaceutical Chemistry Department, Faculty of Pharmacy, Cairo University, Kasr El-Aini St., Cairo 11562, Egypt*

**Keywords:** vildagliptin, pioglitazone hydrochloride, metformin hydrochloride, reversed-phase liquid chromatography, tablets

## Abstract

Two reversed-phase liquid chromatographic (RP-LC) methods are described for the determination of two binary mixtures of hypoglycemic agents. In the first method, vildagliptin (VDG) was determined in the presence of 3-amino-1-adamantanol (AAD), a synthetic intermediate and impurity of VDG. In the second method, pioglitazone hydrochloride (PGZ) and metformin hydrochloride (MET) were simultaneously determined in their binary mixture. Chromatographic separation in the two methods was achieved on a Symmetry^®^ Waters C18 column (150 mm × 4.6 mm, 5 μm). In the first mixture, isocratic elution using a mobile phase of potassium dihydrogen phosphate buffer pH (4.6) - acetonitrile - methanol (30:50:20, *v/v/v*) at a flow rate of 1 mL min^-1^ with UV detection at 220 nm was performed. In the second method, isocratic elution based on potassium dihydrogen phosphate buffer pH (4.6) - acetonitrile (60:40, *v/v*) at a flow rate of 1 mL min^-1^ with UV detection at 210 nm was performed. Linearity, accuracy and precision were found to be acceptable over the concentration ranges of 5-200 μg mL^-1^, 0.5-3 μg mL^-1^ and 10-150 μg mL^-1^ for VDG, PGZ and MET, respectively. The optimized methods were validated and proved to be specific, robust, precise and accurate for the quality control of the drugs in their pharmaceutical preparations.

## INTRODUCTION

**Figure 1:**
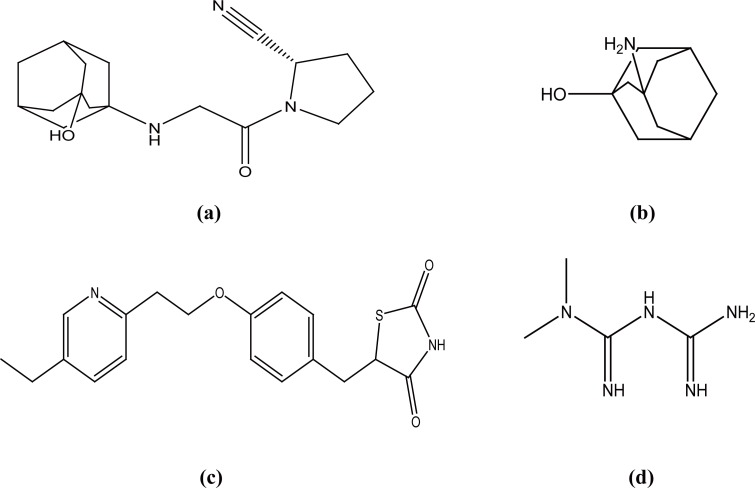
Chemical structures of vildagliptin (a), vildagliptin synthetic intermediate (b), pioglitazone (c) and metformin (d).

Vildagliptin (VDG), S-1-[*N*-(3-hydroxy-1-adamantyl)glycyl]pyrrolidine-2-carbonitrile (Fig. 1a) is an oral hypoglycemic drug of the dipeptidyl peptidase-4 (DPP-4) inhibitor class (1). DPP-4 inhibitors represent a new therapeutic approach to the treatment of type 2 diabetes (2). Literature survey reveals that only one spectrophotometric method was reported for the determination of VDG by the same authors of the present work (3). 3-Amino-1-adamantanol (AAD) (Fig. 1b) has been reported in the synthesis of vildagliptin (1), so it is expected to be an impurity of VDG. Due to lack of published liquid chromatographic methods for VDG, so the aim of the present work was to develop a reversed-phase liquid chromatographic (RP-LC) method that would be suitable for the determination of VDG either alone or in the presence of AAD as an expected impurity.

Pioglitazone hydrochloride (PGZ), ((*RS*)-5-(4-[2-(5-ethylpyridin-2-yl) ethoxy] benzyl)thiazolidine-2,4-dione (Fig. 1c) is one of the thiazolidinedione hypoglycemic agents. Metformin hydrochloride (MET), N,N-dimethylimidodicarbonimidic diamide (Fig. 1d) is a biguanide hypoglycemic drug that is regarded as the main compound in mixed therapies of oral hypoglycemics. Literature survey reveals that MET and PGZ have been simultaneously determined by a few methods, including spectrophotometry (4), HPLC (5, 6), LC/MS (7). Besides, some methods have been reported for determination of MET in mixtures including LC/MS/MS (8) and HPLC (9-12). This study presents the determination of PGZ and MET in binary mixture using HPLC-UV detection. In spite of the similarity of the applied mobile phase with a previous work (5), the minor changes in the mobile phase and the pH proved to shorten the time of the analysis with good peak shapes and good resolution between peaks of PGZ and MET.

## EXPERIMENTAL

### Instrumentation

The HPLC system consisted of a Schimadzu LC-20 AT Liquid Chromatograph (Japan) using a Symmetry^®^ Waters C18 column (150 mm × 4.6 mm, 5 μm) (Ireland). The system was equipped with a UV-visible detector (SPD-20A, Japan) and an autosampler (SIL-20A, Schimadzu, Japan). An Elma S100 ultrasonic processor model KBK 4200 (Germany) was used for the degassing of the mobile phases.

### Reagents and reference samples

Pharmaceutical grade VDG, certified to contain 99.70% and Galvus^®^ tablets nominally containing 50 mg VDG per tablet (batch No. V6498) were kindly supplied from Novartis Europharm limited company (London, U.K.). Pharmaceutical grade PGZ, certified to contain 99.85% and Pioglumet^®^ tablets nominally containing 16.5 mg of PGZ and 850 mg of MET per tablet (batch No. 1001382) were kindly supplied by El Razy Pharma (Ismailia, Egypt). Pharmaceutical grade MET, certified to contain 99.79% was kindly supplied by Chemical Industries Development (CID) Co. (Giza, Egypt). Methanol (HiPerSolv for HPLC), Acetonitrile (HiPerSolv), potassium dihydrogen phosphate and orthophosphric acid (85%) were obtained from VWR Chemicals (Pool, England). Bi-distilled water was produced in-house (Aquatron Water Still, A4000D, UK). Membrane filters 0.45 μm from Teknokroma (Barcelona, Spain) were used. All other chemicals and reagents used were of analytical grade unless indicated otherwise. Standard stock solutions of each drug (1 mg mL^-1^) were prepared by dissolving 100 mg of the drug in methanol in a 100 mL volumetric flask and then completed to volume with methanol. Then required concentrations were prepared by serial dilutions with methanol of these stock solutions.

### Chromatographic conditions

Chromatographic separation was achieved on a Symmetry^®^ Waters C18 column (150 mm × 4.6 mm, 5 μm). In the first mixture (HPLC 1), isocratic elution using a mobile phase consisting of potassium dihydrogen phosphate buffer pH (4.6) - acetonitrile-methanol (30:50:20, *v/v/v*) with UV detection at 220 nm was performed. In the second mixture (HPLC 2), isocratic elution based on potassium dihydrogen phosphate buffer pH (4.6)-acetonitrile (60:40, *v/v*) with UV detection at 210 nm was performed. The buffer solution was filtered through 0.45 μm membrane filter and degassed for 30 min in an ultrasonic bath prior to use. The mobile phase was pumped through the column at a flow rate of 1 mL min^-1^. Analyses were performed at ambient temperature and the injection volume was 25 μL.

### Samples’ preparation

Twenty tablets of each pharmaceutical preparation were weighed. An accurately weighed amount of the finely powdered Galvus^®^ tablets equivalent to 100 mg of VDG was made up to 100 ml with methanol. An accurately weighed amount of the finely powdered Pioglumet^®^ tablets equivalent to (16.5 mg) PGZ and (850 mg) MET were made up to 100 ml with methanol. The solutions were filtered followed by serial dilution to the required concentrations for each experiment.

### Procedure

#### • Linearity and repeatability

**For HPLC 1.** Accurately measured aliquots of working standard solutions equivalent to 50-2000 μg VDG were transferred into a series of 10 mL volumetric flasks and then completed to volume with methanol. A volume of 25 μL of each solution was injected into the chromatograph. The chromatographic conditions mentioned under 2.3. including the mobile phase at a flow rate 1 mL min^-1^, detection at 220 nm and run time program for 10 min were adjusted. A calibration curve for VDG was obtained by plotting area under the peak (AUP) against concentration (C). The repeatability of the method was assessed by analyzing a mixture containing 100 μg mL^-1^ of VDG and 30 μg mL^-1^ of AAD (*n*=6). The precision (%R.S.D) was calculated (Table 1).

**Table 1:** System suitability test for the proposed LC method (HPLC 1) for the determination of vildagliptin in binary mixture with its impurity

Item	VDG	VDG impurities

N	1872	3136
R	2.92	
T	1.1	1.01
RSD% of 6 injections		
Peak area	0.67	0.82
Retention time	0.18	0.23

**For HPLC 2.** Accurately measured aliquots of working standard solutions equivalent to 5-30 μg PGZ and 100-1500 μg MET were separately transferred into two series of 10 mL volumetric flasks and then completed to volume with methanol. A volume of 25 μL of each solution was injected into the chromatograph. The chromatographic conditions mentioned under 2.3. including the mobile phase at a flow rate 1 mL min^-1^, detection at 210 nm and run time program for 7.5 min were adjusted. A calibration curve for each compound was obtained by plotting area under the peak (AUP) against concentration (C). The repeatability of the method was assessed by analyzing a mixture containing 1.65 μg, 85 μg of PGZ and MET, respectively (*n*=6). The precision (%R.S.D) for each compound was calculated (Table 2).

**Table 2:** System suitability test for the proposed LC method (HPLC 2) for the simultaneous determination of pioglitazone and metformin in binary mixture

Item	PGZ	MET

N	3413	1681
R	4.2	
T	1.00	1.06
RSD% of 6 injections		
Peak area	0.85	0.73
Retention time	0.26	0.39

#### • Assay of drugs in laboratory prepared mixtures and in pharmaceutical dosage forms

**For HPLC 1.** The procedure mentioned under 2.5.1.1 was repeated using concentrations equivalent to 45-130 μg mL^-1^ VDG and 15-40 μg mL^-1^ AAD (10% to 30% of VDG, *w/w*). For the determination of the examined drug in Galvus^®^ tablets, the sample solution prepared under 2.4 was serially diluted to prepare solutions equivalent to 35-120 μg mL^-1^ of VDG and then injected in triplicates. The concentrations of the examined drug were calculated using calibration equation.

**For HPLC 2.** The procedure mentioned under 2.5.1.2 was repeated using laboratory prepared mixtures equivalent to 1-2.8 μg mL^-1^ PGZ and 55-145 μg mL^-1^ MET. The concentration ratios of PGZ to MET in the mixtures were 1.5-2.5%, *w/w*. For the determination of the examined drugs in Pioglumet^®^ tablets, the sample solution prepared under 2.4 was serially diluted to prepare solutions equivalent to 0.49-2.43 and 25-125 μg mL^-1^ of PGZ and MET, respectively; and then injected in triplicates. The concentrations of the examined drugs were calculated by the calibration equations.

## RESULTS AND DISCUSSION

HPLC greatly reduces the analysis time and allows for the determination of many individual components in a mixture using one single procedure (13). No previous method was reported for the LC determination of VDG. Thus, the aim of this work was to develop a new, simple, accurate, reproducible and sensitive LC method for the determination of VDG either alone or in the presence of the intermediate of its synthesis (AAD) (HPLC 1).

Alternatively, a previous work (5) described a reversed-phase LC method for the simultaneous determination of PGZ and MET. A Gemini C18 column (150 × 4.6 mm, 5 μ) was used with a mobile phase containing a mixture of acetonitrile and ammonium acetate buffer (pH 3) in the ratio of 42:58. The flow rate was 0.3 mL min^-1^ and effluents were monitored at 255 nm and eluted at 5.17 min (MET) and 8.1 min (PGZ). Calibration curves were plotted with a range from 0.5-50 μg mL^-1^ for MET and 0.3-30 μg mL^-1^ for PGZ. Upon investigation of such chromatographic conditions, one can first notice the flow rate (0.3 mL min^-1^) is low as the typical LC flow rate should be from 0.5 to 1 mL min^-1^ (14). Besides, ranges of the calibration are not suitable to the ranges of PGZ and MET in tablets. On the other hand, our method has the advantage of a shorter retention times with better sensitivity with a reasonable flow rate (1 mL min^-1^) and suitable linearity ranges for the two drugs.

### Methods’ development

**Figure 2:**
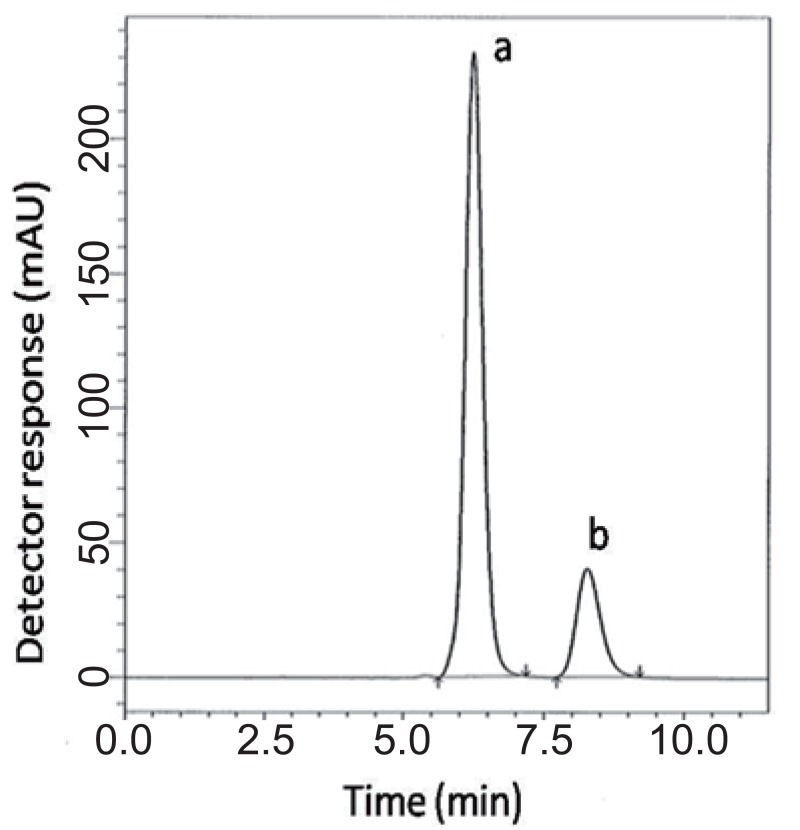
A typical LC chromatogram of 25 μL injector of synthetic binary mixture of (a) vildagliptin (100 μg mL^-1^) and (b) vildagliptin impurity (30 μg mL^-1^).

**For HPLC 1.** Various isocratic mobile phase systems at different pH values were attempted. Isocratic elution based on potassium dihydrogen phosphate buffer pH (4.6) - acetonitrile - methanol (30:50:20, *v/v/v*) was applied. Minimum retention times were obtained at a flow rate 1 mL min^-1^. The UV detector was operated at 220 nm where good detector sensitivity was achieved. The retention times were 6.2 and 8.2 min for VDG and AAD, respectively (Fig. 2).

**Figure 3:**
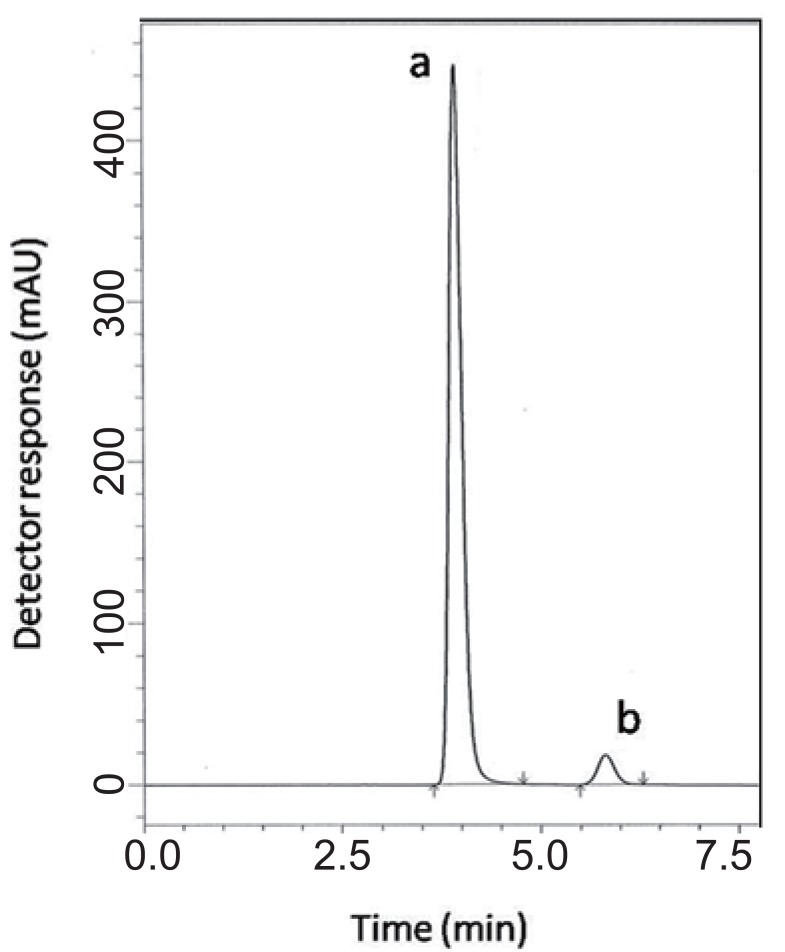
A typical LC chromatogram of 25 μL injector of synthetic binary mixture of (a) metformin (150 μg mL^-1^) and (b) pioglitazone hydrochloride (2.9 μg mL^-1^).

**For HPLC 2.** Various reversed-phase columns, isocratic mobile phase systems were attempted. Isocratic elution based on potassium dihydrogen phosphate buffer pH (4.6)-acetonitrile (60:40, %*v/v*) was applied. Minimum retention times were obtained at a flow rate 1mL min^-1^. The UV detector was operated at 210 nm where good detector sensitivity was achieved. The retention times were 5.90 and 4.07 min for PGZ and MET, respectively; as presented in Fig. 3.

### System suitability tests

According to USP (15), system suitability tests are an integral part of liquid chromatographic methods in the course of optimizing the conditions of the proposed method. In the two proposed LC methods, system suitability tests are used to verify that resolution and reproducibility were adequate for analysis performed. Different parameters affecting the chromatographic separation were studied. The parameters of this test are column efficiency (number of theoretical plates), tailing of chromatographic peak, peak resolution factor, and repeatability as % R.S.D of peak areas for six injections and reproducibility of retention times. The results of these tests are listed in Tables 1-2.

### Methods’ validation

**Linearity.** Linearity was studied for VDG, PGZ and MET. A linear relationship between area under the peak (AUP) and component concentration (C) was obtained. The regression equations were also computed. The linearity of the calibration curves were validated by the high value of correlation coefficients. The analytical data of the calibration curves including standard deviations for the slope and intercept (S_b_, S_a_) are summarized in Tables 3-4.

**Table 3:** Results obtained by LC method (HPLC 1) for the determination of vildagliptin in binary mixture with its impurity (3-amino-1-adamantanol)

Item	vildagliptin

Retention time	6.3
Wavelength of detection	220 nm
Range of linearity	5-200 μg.ml^-1^
Regression equation	Area × 10^-5^ = 0.5438 C_μg/ml_ - 0.1127
Regression coefficient (r^2^)	0.9998
LOD μg.ml^-1^	1.46
LOQ μg.ml^-1^	4.87
S_b_	3.5 × 10^-3^
S_a_	3.1 × 10^-2^
Confidence limit of the slope	0.5438 ± 1.69 × 10^-2^
Confidence limit of the intercept	-0.1127 ± 3.94 × 10^-4^
Standard error of the estimation	0.176
Intraday	
% R.S.D	0.26-0.97
Interday	
% R.S.D	0.43-1.61
Drug in laboratory mixture	100.01 ± 1.75
Drug in dosage form	99.74 ± 1.03
Drug added	100.50 ± 1.13

**Table 4:** Results obtained by LC method (HPLC 2) for the simultaneous determination of pioglitazone and metformin in binary mixture

Item	Pioglitazone	Metformin

Retention time	5.90	4.07
Wavelength of detection	210 nm	210 nm
Range of linearity	0.5-3 μg.ml^-1^	10-150 μg.ml^-1^
Regression equation	Area × 10^-4^ = 11.7832 C_μg/ml_ - 0.0118	Area × 10^-6^ = 0.0409 C_μg/ml_ + 0.0418
Regression coefficient (r^2^)	0.9997	0.9999
LOD μg.ml^-1^	0.06	1.95
LOQ μg.ml^-1^	0.17	5.91
S_b_	1.32 × 10^-2^	6.7 × 10^-4^
S_a_	2.26 × 10^-2^	6.5 × 10^-2^
Confidence limit of the slope	11.7832 ± 26.63 × 10^-2^	0.0409 ± 0.27 × 10^-2^
Confidence limit of the intercept	-0.0118 ± 1.56 × 10^-4^	0.0418 ± 0.28 × 10^-4^
Standard error of the estimation	0.239	0.069
Intraday		
% R.S.D	0.26-0.59	0.13-0.43
Interday		
% R.S.D	0.37-1.75	0.62-1.26
Drug in laboratory mixture	99.99 ± 1.50	100.40 ± 1.60
Drug in dosage form	99.52 ± 1.38	100.73 ± 1.55
Drug added	100.13 ± 1.87	100.33 ± 1.33

### Accuracy.

#### • For HPLC 1

Accuracy of the results was calculated by % recovery of 5 different samples of VDG in laboratory prepared mixtures with AAD also by standard addition technique for Galvus^®^ tablets. The results obtained including the mean of the recovery and standard deviation are displayed in Table 3.

#### • For HPLC 2

Accuracy of the results was calculated by % recovery of 5 different samples of the laboratory prepared mixtures of PGZ and MET and also by standard addition technique for Pioglumet^®^ tablet. The results obtained including the mean of the recovery and standard deviation are displayed in Table 4.

### Precision.

#### • For HPLC 1

The repeatability of the method was assessed by analyzing a mixture containing 100 μg mL^-1^ of VDG and 30 μg mL^-1^ of AAD (*n*=6). The values of the precision (% R.S.D)of repeatability and inter-day and intra-day precision (using 3 different concentrations in triplicates for three days) are displayed in Tables 1 & 3.

#### • For HPLC 2

The repeatability of the method was assessed by analyzing 1.65 μg mL^-1^ of PGZ and 85 μg mL^-1^ of MET (*n*=6). The values of the precision (% R.S.D) of repeatability and inter-day and intra-day precision (using 3 different concentrations in triplicates for three days) are displayed in Tables 2 & 4.

### Specificity.

**Figure 4:**
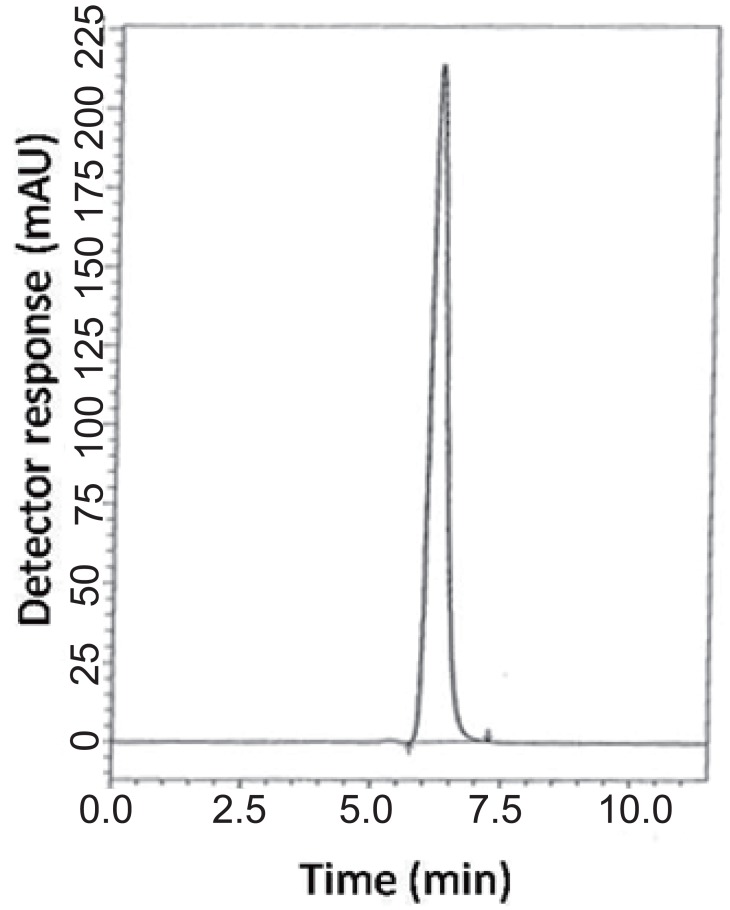
A typical LC chromatogram of 25 μL injector of 100 μg mL^-1^ Galvus^®^ sample solution.

**Figure 5:**
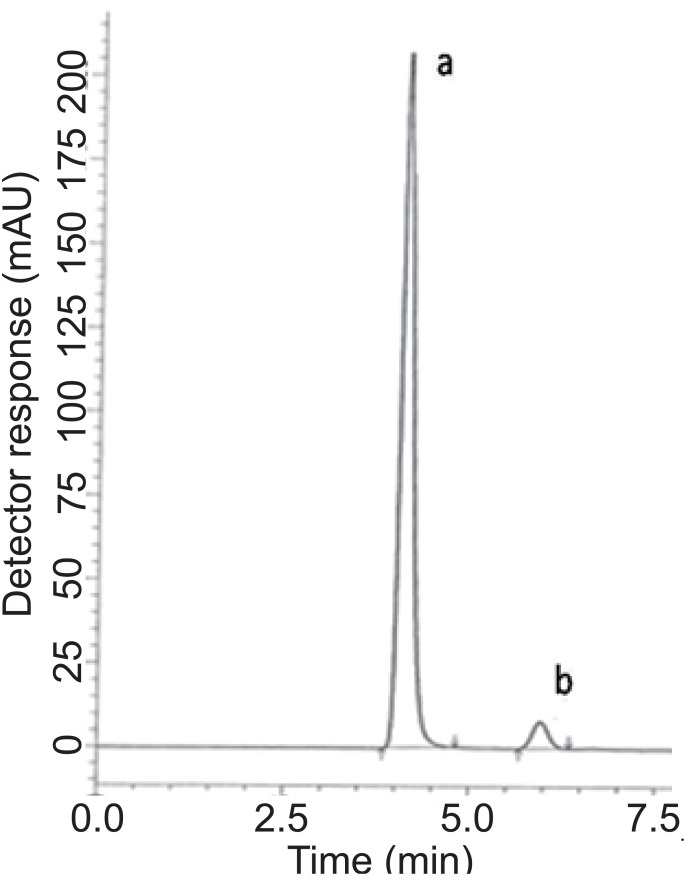
A typical LC chromatogram of 25 μL injector of Pioglumet^®^ sample solution, (a) metformin (85 μg mL^-1^) and (b) pioglitazone hydrochloride (1.65 μg mL^-1^).

Specificity is the ability of the analytical method to measure the analyte response in the presence of interferences including degradation products and related substances. In the present work, specificity was checked by analyzing VDG with AAD and PGZ with MET in laboratory prepared mixtures. Good resolution and absence of interference between drugs being analyzed are shown in Fig. 2-3. Besides, the chromatograms of the pharmaceutical formulation samples were checked for the appearance of any extra peaks. No chromatographic interference from any of the excipients was found at the retention times of the examined drugs (Fig. 4 & 5). In addition, the chromatograms of the drugs in the samples’ solutions were found identical to the chromatograms received by the standard solutions at the wavelengths applied. Moreover, good results were obtained for the determination of the cited drugs in the two dosage forms, Tables 3-4. These results confirm the absence of interference from other materials in the pharmaceutical formulations and therefore confirm the specificity of the two proposed methods.

### Robustness.

#### • For HPLC 1

Robustness was performed by deliberately changing the chromatographic conditions. The most important parameter to be studied was the resolution factor between the two peaks of VDG and AAD. The flow rate of the mobile phase was changed from 1 mL min^-1^ to 0.8 mL min^-1^ and 1.2 mL min^-1^, where resolution factors obtained were 2.92, 2.71 and 2.65 respectively. The organic strength was changed by ± 2% where resolution factors obtained were 2.92, 2.63 and 3.15 respectively. Finally, the value of pH of the phosphate buffer was varied from 4.6 to 4.5 and 4.7, where resolution factors obtained were 2.92, 2.54 and 3.22 respectively. As can be seen from these results, good values of the resolution factor were obtained for all these variations, indicating good robustness of the proposed LC method.

#### • For HPLC 2

The most important parameter to be studied was the resolution factor between the two peaks of PGZ and MET. The flow rate of the mobile phase was changed from 1 mL min^-1^ to 0.8 mL min^-1^ and 1.2 mL min^-1^, where resolution factors obtained were 4.20, 4.51 and 4.63 respectively. The organic strength was changed by % ± 2 where resolution factors obtained were 4.20, 3.86 and 3.91 respectively. Finally, a value of pH of the phosphate buffer was varied from 4.6 to 4.5 and 4.7, where resolution factors obtained were 4.20, 3.81 and 3.71 respectively. As can also be seen from these results, good values of the resolution factor were obtained for all these variations, indicating good robustness of the proposed LC method.

**Limit of detection and limit of quantification.** Limit of detection (LOD) which represents the concentration of analyte at S/N ratio of 3 and limit of quantification (LOQ) at which S/N is 10 were determined experimentally for the proposed methods and results are given in Tables 3-4.

**Statistical analysis.** Statistical analysis of the results obtained by the proposed methods and the reference methods for each drug were carried out by “SPSS statistical package version 11”. The significant difference between the reference methods and the described methods was tested by one way ANOVA (F-test) at *p*=0.05 as shown in Tables 5-6. The test ascertained that there was no significant difference among the methods.

**Table 5:** Statistical comparison between the results of the proposed LC method (HPLC 1) and the reference method for the determination of vildagliptin

Statistical Term	Reference Method^b^	Proposed method

Mean	100.01	100.008
S.D.±	0.99	1.75
S.E. ±	0.44	0.78
%RSD	0.99	1.75
n	5	5
V	0.98	3.06
t (a2.306)		0.002
F (a6.39)		0.32

a Figures in parentheses are the theoretical t and F values at (*p*=0.05);

b Reference method: aliquots of standard solutions in distilled water containing 5–25 μg/ml VDG were measured at 210 nm using water as a blank (3).

**Table 6:** Statistical comparison between the results of the LC method (HPLC 2) and the reference methods for the determination of pioglitazone and metformin

Statistical Term	Reference Method for PGZ^b^	HPLC method	Reference Method for MET^c^	HPLC method

Mean	100.2	99.99	100.4	100.398
S.D.±	1.15	1.50	0.28	1.60
S.E. ±	0.51	0.67	0.13	0.72
%RSD	1.15	1.50	0.28	1.59
n	5	5	5	5
V	1.32	2.25	0.08	2.56
t (a2.306)		0.25		0.003
F (a6.39)		0.59		0.03

a Figures in parentheses are the theoretical t and F values at (*p*=0.05);

b Reference method for the HPLC determination of pioglitazone (4);

c Reference method for the spectrophotometric determination of metformin in the indian pharmacopeia (16).

## CONCLUSION

The proposed LC methods have the advantages of simplicity, precision, accuracy and convenience for the separation and quantization of VDG in combination with AAD, and also separation and simultaneous determination of PGZ in combination with MET. The two methods can be applied for the determination of the cited drugs in pharmaceutical dosage forms. The two methods were validated showing satisfactory data for all the method validation parameters tested. The developed methods can be conveniently used by quality control laboratories.
